# On Diseases of the Liver

**Published:** 1846-03

**Authors:** 


### REVIEWS.

**Art. III.**—*On Diseases of the Liver.* By George Budd, M.D., F.R.S., Professor of Medicine in King's College, London; and Fellow of Caius Cambridge. *With colored Plates and numerous Woodcuts.*—Philadelphia: Lea & Blanchard. 1846. 12mo. pp. 392.

[concluded.]

The subjects of this treatise are on every account so important, a knowledge of them is so necessary to the practitioner, and they are here treated with so much clearness and ability, that we are sure our readers will not be displeased at a continuation of our notice of the volume. In our last number we left our author on the *causes of abscess of the liver*, the most obvious of which, though by no means a frequent one, is a blow or other mechanical injury. The second, and, as he holds, far more frequent cause, *suppurative inflammation of some vein*, we announced, but were obliged to close our article without reciting any of the proofs adduced in support of the position. As we then said, the point strikes us as satisfactorily made out, and some of the evidence in its favor we will now proceed to give.

It is known that collections of pus in the lungs, liver, joints, or between the muscles, frequently follow severe injuries or surgical operations—that they form rapidly, and, with very slight local symptoms, often terminate fatally in a few days. So when mercury is injected into the crural vein of a dog, the animal, after a day or two, becomes feverish, is affected with cough and difficulty of breathing, of which he dies; and on examination after death is found with small indurated masses and circumscribed abscesses in his lungs, each of which contains in the centre a small globnle of mercury. In such cases the foreign matter is arrested in the capillary vessels of the lungs, for the pus globules are much larger than the globules of the blood; irritation follows, and a deposit of matter is the result. The lungs are the organs most likely to suffer from such an experiment, or from purulent phlebitis consequent on injury of the head or limbs, for the obvious reason that the irritating matters are conveyed directly to them, and may be wholly detained in their capillaries. In the latter case abscesses would be found only in the lungs. But it often happens that the liver becomes the recipient of a part of the pus, and then it too is the seat of scattered abscesses.

It would appear from some observations by Cruveilhier, that such abscesses are most apt to ensue upon injuries involving bones, inflammation of the veins in the interior of bones being more likely to cause them, than inflammation of the veins of other textures. He placed a globule of mercury in the medullary cavity of the femur of dogs, and never failed to find it scattered through the lungs, each globule the centre of a small abscess. Injuries of the head, it has been long noticed, are especially liable to be followed by hepatic abscesses, and in the above circumstances we have a satisfactory explanation of the fact. In like manner cancer of the breast may be the source of cancerous tumors in the lungs and liver, the cancer-cells, like the pus-globules, being disseminated through the veins. In view of all this our author thinks that—

"It may be considered established, that the abscesses which form in the liver and other organs, after surgical operations and injuries of the head or limbs, are owing to suppurative inflammation of a vein, and the consequent contamination of the blood by pus. The globules of pus, mingled with the blood, are conveyed to the capillary vessels of the lungs, and, it would seem, by becoming mechanically arrested there, excite circumscribed inflammation and abscess. If any of the globules pass through the capillaries of the lungs to the left side of the heart, they are sent in the arterial current to other organs, and becoming arrested in the capillaries of these organs, excite, as in the lungs, inflammation of limited extent, rapidly passing on to abscess."

Thus far the author has alluded only to inflammation of those veins which return their blood immediately to the vena cava, in which case the pus must pass through the capillaries of the lungs before it can be sent to other organs. But there are veins in the body of a different destination—those that go to form the vena portæ. One of these becoming inflamed, the pus goes not to the lungs first to be detained in them, but to the liver where it sets up suppurative action. Cruveilhier found it so; for when he injected mercury into one of the veins that feed the vena portæ, it was stopped in the liver, and became the source of abscesses there, just as it led to the same in the lungs when injected into the crural vein.

"He injected mercury into one of the mesenteric veins of a dog. At the end of twenty-four hours, the dog died, and the surface of the liver was found sprinkled with small spots of a deep red color, which extended four or five lines into its substance. In the centre of each of these red masses was a small globule of mercury."

Injury of any of these veins is often followed by abscess of the liver. Cruveilhier relates a case where it was consequent upon repeated attempts to return a prolapsed rectum. It has been known to result from operations on the rectum and for strangulated hernia. Hence our author concludes there is a third, and much more frequent cause of abscess of the liver than either of those mentioned: namely, *ulceration of the intestines*, *stomach*, *gall-bladder*, *or ducts*, all of which return their blood through the liver.

A connexion between abscess of the liver and dysentery has long been noticed, but in the opinion of Dr. Budd they are associated far more frequently than has been generally imagined. Thus, in twenty-nine cases recorded by Annesley, in India, there were twenty-one in which ulcers were discovered in the large intestine; in two others the bowel was otherwise diseased, and in the remaining cases, our author thinks it probable, ulceration of the intestines existed but was overlooked. Of thirteen cases in which he made the autopsy, nine presented ulcers either in the large or small intestines, or in the stomach. Andral and Louis, without suspecting any connexion between ulcerated intestine and abscess of the liver, of sixteen cases noticed ulcers in the bowels, stomach, or gall bladder in eight, or one half. Of the others, one was caused by a blow, and four seemed the consequence of phlebitis. It is worthy of remark that in the latter five cases no ulcers were found in the stomach, intestines, or gall-bladder; deducting these cases attributable to other causes we have eleven left, which must be referred to ulceration in one or the other of these parts.

The author multiplies facts tending to the same point, but we do not deem it necessary to add to the testimony in favor of his position. Admitting then that abscess of the liver, where not dependent upon mechanical violence, is for the most part connected with ulceration of some part of the alimentary canal, the question may arise, is this from a spread of inflammation to the liver, or from some contamination of the portal blood? Obviously, Dr. Budd maintains, from the latter cause, and he goes on to say—

"This may be either by *pus*, formed by suppurative inflammation of one of the small intestinal veins; or by matter of other kind resulting from softening of the tissues; or by the fetid gaseous and liquid contents of the large intestine in dysentery, which must he absorbed and conveyed immediately to the liver. It seems probable, that contamination of the first kind usually gives rise to small scattered abscesses; of the last, to diffuse inflammation, and a larger, perhaps single, collection of pus. If the morbid matter be such, that it does not mix readily with the blood—as globules of pus or mercury—it will cause small, circumscribed abscesses, the rest of the liver being healthy. If, on the contrary, the morbid matter be readily diffusible in the blood, all the blood will be vitiated, and diffuse inflammation result."

But, he holds, it is not every form of ulceration of the stomach and intestines, that gives rise to abscess of the liver. It is not apt to result from the ulceration attendant upon typhoid fever; nor from ulceration of the duodenum after burns, or of the intestines in phthisis. It seems to occur chiefly in conjunction with the sloughing ulceration in acute dysentery; "and with chronic ulcers attended with thickening and induration of the sub-mucous areolar tissue."

Not more than one of the causes assigned by our author for hepatic abscess is found, it would appear, in the same subject, and this circumstance is strongly confirmatory of his view of the origin of that malady. Where the abscess could be traced to a blow, or to suppurative inflammation of some vein that returns its blood immediately to the vena cava, there were no ulcers in the stomach, bowels, or gall ducts. And when ulcers were found in the intestines, they were not present in the stomach or gall-bladder.

Congestion, spirit-drinking, and the many other causes to which the abscess of the liver has been ascribed by authors, Dr. Budd contends, do not lead to that result. Ardent spirits excite inflammation, but do not produce abscess of the liver, according to his observation.

The size of these abscesses is often very great,. Dr. B. mentions one which contained two quarts. He quotes a case from Annesley in which an abscess in the liver contained ninety ounces of matter; and one from Dr. Inman in which the quantity of matter was thirteen pints. As to the quality of the matter, he says:

"The matter in an hepatic abscess is usually white or yellowish; and is free from odour, unless when in close proximity to the lung, where it sometimes becomes decomposed and fetid, from the admission of air."

But not to dwell longer on the causes and anatomy of this form of hepatic ailment, we turn next to the *symptoms*, respecting which our author holds the following discouraging language:

"In most works on medicine, these have been described as being much more uniform than they really are. A picturesque group is sketched, which it seems very easy to identify; but in actual practice, it is far otherwise. The physicians who have had most experience in this disease, confess their inability, in many cases, to distinguish it from other diseases of the liver; and in some, even to pronounce that the liver is the seat of disease at all. Here, as in the diseases of other internal organs, our diagnosis will be much aided by a knowledge of the circumstances under which the disease arises. This knowledge will make us observant of symptoms which would otherwise escape our notice, and will enable us to interpret them rightly."

This obscurity in the symptoms—this absence of all symptoms, in many cases, directly referrible to the liver—he attributes to the circumstance, that "suppurative inflammation is generally partial, and often involves only the *substance* of the liver, the natural sensibility of which is slight."

Among the symptoms—as dry cough, pain in the right shoulder, pain and tension in the right hypochondrium, and the general characteristics of inflammatory fever, there is one to which he attaches special importance;—that is, "permanent rigidity of the muscles of the abdominal parietes, but especially *of the right rectus muscle.*" The following case illustrates the value of this symptom, and deserves to be carefully noted:

"In the autumn of 1837, a sailor, 29 years of age, was admitted into the Dreadnought, immediately on his arrival from Calcutta. He was much emaciated, and stated that he had been ill thirty days of fever, and that during the last ten days, he had vomited every thing he had taken. His belly was much drawn in, and the parietes were extremely rigid, but there was no tenderness on pressure. He was somewhat thirsty, but afraid to drink, on account of the vomiting it immediately excited. My impression was that his disease was gastritis, and I prescribed for him accordingly. The symptoms increased, and at the end of a fortnight he could be got to take little besides toast and water, which he sipped rather than drank. He died about a month after his admission to the Dreadnought. The stomach was found apparently sound, but the liver was the seat of a large abscess, the presence of which was not even suspected."

Abscess of the liver may discharge itself in various ways. Thus, it may burst into the cavity of the peritoneum, if situated on the surface of the liver; or, adhesion forming, it may discharge itself through the abdominal parietes; if on the upper part of the liver, it may perforate the diaphragm and burst into the cavity of the pleura, or, more favorably, may make its way by adhesion through the lungs; or, finally, it may be discharged through the intestinal canal, adhesions having formed between the edge of the liver and the stomach, duodenum, or large intestine.

This malady is of a protracted character, even when not fatal, which is generally the case. The hepatic tissue and the hard gristly substance surrounding the abscess cannot contract; the cavity consequently remains unclosed, and continues to be filled with pus. It is, therefore, a matter of less consequence than would at first appear which direction the pus may take; for if the abscess cannot heal death must eventually occur, although in one case it may be longer averted than in another.

The treatment of suppurative inflammation of the liver, it follows from what has been said, is exceedingly unsatisfactory. If it is caused by phlebitis consequent on a wound or injury of the head or limbs, but little can be done. The general contamination of the venous blood by pus leads to inflammation in the lungs, and perhaps in other parts of the body—a condition of things for which we have no remedy.

Where its origin is in a blow we have more to expect from treatment. Active measures employed early may prevent the formation of matter. Mercury is one of the remedies which have been resorted to in all forms of hepatic disease; but its efficacy in inflammation of that organ is questioned by Dr. Budd; he considers that it is "peculiarly unsuited to suppurative inflammation of the liver."

In some cases there seems to arise a necessity for opening the abscess, but great danger attends the operation. A distended gall-bladder may be mistaken for an abscess—a mistake which would be almost immediately fatal to the patient.

This, however, may be avoided. The gall-bladder distended by bile is globular, circumscribed, hard, and equally resisting in every part; an abscess is soft and fluctuating at its summit, with a hard, resisting base, and is not so well defined.

A second and more serious danger grows out of the circumstance that the liver does not always become adherent to the abdominal parietes; in which case an incision would empty the pus into the peritoneal sac, and occasion sudden death. The directions of the author under circumstances of such uncertainty are the best that could be given.

"I would, then, never recommend opening an abscess of the liver, unless assured by circumscribed œdema, or a slight blush on the skin, that union had taken place between the integument and abscess. When these signs are wanting, and the skin has its natural appearance and color, we can never be sure that adhesions have formed, and if we thrust a knife into the abscess, we run the risk of discharging the matter into the cavity of the peritoneum."

He gives the method recommended by Dr. Graves for obviating this danger. This consists in laying open the abdominal muscles down to the peritoneum, filling up the wound with lint, and then waiting for adhesive inflammation to unite the liver to the side, and for the abscess to open of itself.—Thus we provide against two accidents; but the author enumerates others—as the entrance of air into the wound, which may lead to gangrene, and speedily carry off the patient; and, since the abscess when opened will continue to discharge until the system is exhausted by the protracted suppuration, he concludes that the patient only dies the earlier for our meddling.

*Gangrenous inflammation of the liver* occurs rarely, but cases of it are related by the author. In one, it followed gangrene of the toes from cold, and seems to have been excited by it; it was accompanied by gangrene in the lungs also. Gangrene, however excited, in one part, has a tendency, in the words of our author, "to cause gangrene in other parts remote from it and not subject to the same influence." He supposes this is explained by the chemical law, that matter in a state of change tends to bring whatever parts it may touch into the same state of transformation. He quotes Rokitansky to the effect, that he has several times observed gangrene of the liver in connexion with gangrene of the lung, and never without gangrene of some other part.

The section which follows treats of "Adhesive inflammation of the capsule and of the substance of the liver, Cirrhosis, and other forms of inflammation of the substance of the liver." Various causes may excite these inflammations, but the most frequent is the use of spirituous liquors. The case of ascites and vomiting of blood, reported in this number of the Journal is in point. Dr. Budd relates cases marked by the same symptoms. These forms of liver-disease are most frequent in large towns where the poorer classes drink hard; the granular and hob-nail liver, called by the French cirrhosis, is familiarly termed the *gin-drinkers'* liver by English practitioners, so generally may it be traced to indulgence in that potation.

We pass over the symptoms of cirrhosis minutely given by the author, and quote from his remarks on its diagnosis.

"In the early stage of cirrhosis, the symptoms are often few, and present no distinctive, and, to common eyes, no alarming characters—so that it is only by considering the circumstances in which they arise that we are led to perceive their true significance. Slight sallowness of complexion, a dull pain or some degree of tenderness in the right hypochondrium, with occasional feverishness, in a person above the age of thirty, who has been long in the habit of drinking spirits to excess, are almost conclusive evidence of the existence of cirrhosis, even before there is any direct proof that the circulation through the liver is impeded. The symptoms, in themselves, may be slight, but knowledge of the habits of the patient enables us to regard them as tokens of organic and incurable disease. Here, as in so many other cases, it is only by knowing the causes of the disease, or the circumstances under which it usually occurs, that we become watchful of its earliest tokens, and perceive, as it were, the shadows that, in coming, it casts before it.

"When by the progress of the disease the circulation through the liver is so impeded as to cause ascites, the diagnosis is much more easy. The cases most difficult to distinguish from it are those in which ascites is associated with great enlargement of the spleen. It now and then happens that a man is brought into our London hospitals with great ascites, and most of the other symptoms of cirrhosis. After a time the ascites diminish, and the spleen is found enormously enlarged. The ascites may completely disappear, and the patient regain health enough for his former pursuits. I have met with three cases of this kind, but have never ascertained by dissection what the disease really is. The spleen is much enlarged, which it is not in cirrhosis, and the complexion may not be sallow, but in other respects the characters of the two diseases are almost identical."

The previous habits of the patient will afford a clue to the nature of his complaint. While peritonitis, which has some symptoms in common with cirrhosis, attacks persons of all modes of life, only the intemperate are apt to be afflicted with cirrhosis. So too of cancer; it is found in the higher classes, and in the temperate; cirrhosis is seldom seen, except among those who have suffered privations, and drunk spirits to excess.

The treatment to be useful must be early applied. The best remedies are then cupping over the region of the liver, with saline medicines, and low diet. But as hard drinkers do not bear bleeding well, the cupping must not be carried too far, or delirium tremens may supervene. When pushed as far as is deemed safe, it may be followed with advantage by a blister. Mercury and iodide of potassium should follow, the former being carried to the point of slightly touching the gums.

Inflammation of the veins of the liver is another form of hepatic disorder treated of in this volume. It may be seated in the vena portæ, or the hepatic veins, and in the trunks or the branches. A blow may excite it, or it may follow suppuration in other parts, as that which results from injuries of the brain. The author supposes that inflammation of the hepatic veins is of more frequent occurrence than is generally believed, being overlooked in post mortem dissections.

The gall-bladder and ducts are subject to various forms of inflammation, styled by our author catarrhal, suppurative, croupal, and ulcerative. These several modifications depend upon the causes which excite the inflammation. The first, he thinks, is not uncommon. It is a mild disease, attended with feverishness, slight pain in the region of the liver, and, if many of the ducts become closed by thickening of their coats, or by the viscid secretion, with slight enlargement of the liver and jaundice. Many of the cases of simple jaundice coming on in healthy persons, and attended with very little pain and fever he ascribes to catarrhal inflammation of the hepatic ducts.

Suppurative inflammation is of a severer form. It sometimes ends in death, cases of which the author reports. Permanent obstruction in the cystic duct is one of the most frequent causes of it.

Croupal inflammation is rare, while ulceration of the gall-bladder is the most common of all the forms of inflammation yet considered. It has been found among the morbid appearances of remittent fever; often it is produced by gall-stones, and seems especially liable to occur in persons in whom the gall-bladder has suffered from former disease. It may lead to various results; as to a discharge of the contents of the gall-bladder into the cavity of the peritoneum, from sloughing of the coats; or to a similar discharge into the duodenum or colon, adhesions having been previously established between them and the gall-bladder; or to abscesses of the liver, as was shown in the chapter on that subject.

The treatment in all forms of hepatic inflammation is materially the same. Early local depletion is the main remedy, and is of the greatest importance. The symptoms, though slight in the beginning, may end in fatal closure of the ducts of the liver, and should be met in the first stage. Our author's words on this point convey a true idea of the importance of a correct diagnosis of the affection and opportune treatment. He says—

"In such cases, our end must be, not so much to relieve the present symptoms, which are often slight, as to prevent those changes of structure, which, slowly it may be, but inevitably, and with much suffering, destroy life. How infinitely valuable, then, is that insight which enables us to see the danger before it is revealed to other eyes, and when alone we can effectually guard against it. This insight we can derive only from knowledge of the circumstances under which these forms of disease occur; knowledge, which gives meaning to symptoms, otherwise vague, and perhaps so slight as to be scarcely regarded."

To local depletion must be added appropriate diet, and the judicious use of mercury. Soda is mentioned as a remedy of value in such cases, and Dr. Budd thinks favorably of it. He also speaks well of taraxacum, as possessing the power of modifying the qualities of the bile.

We come now to another class of hepatic disorders—those which result from *faulty nutrition* of the liver, or *faulty secretion;* among which are enumerated softening of the liver, destruction of the hepatic cells, suppressed secretion of bile, and fatal jaundice.

These affections are less understood than the inflammatory diseases of the liver. They may be divided into two principal groups, the character of one of which is suspension of the secretion of bile; of the other, that the hepatic cells separate from the blood some abnormal matter, which is retained in the liver, adding to its size, and changing its texture and appearance. Fatal jaundice attends this state of the liver. No obstruction to the flow of bile exists; but the structure of the liver is changed; its size is diminished, it is "soft, or flabby, and of a light yellow, or brownish-yellow, or crimson-orange, or some kindred tint."

This condition of the liver may arise from various causes; as poison, malignant fevers, or even mental distress; it is generally soon followed by delirium, or stupor, passing into coma or convulsions. Still it is not necessarily fatal, even after the patient has reached the borders of this state; although it sometimes proves so from mere exhaustion, without delirium, coma, or convulsions. Having cited numerous cases illustrative of all the shades of this affection, the author concludes with this passage:

"I have brought together from different sources the cases related in this chapter, for the sake of showing that the secretion of bile may be suppressed, and the secreting substance of the liver be more or less disorganized, in various circumstances, and without the occurrence of any process that we are warranted in designating *inflammation.* It would seem that this suspension of the secreting process and disorganization of the liver, may result from powerful and depressing emotions; but that it is far more frequently produced by some poison, introduced from without, or generated in the body by faulty assimilation or digestion. It appears, too, that various poisons, —pus, the poison of serpents, perhaps the poison of some forms of fever, and various others,—may alike stop the secretion of the liver, and lead to the same kind of disorganization of its structure, while their other effects on the system are very different. It is probable, too, that in some cases, as in those last related, the disorganization is produced slowly and gradually, and so *without shock;* while in the more terrible forms of disease, of which instances were before given, the disorganization is sudden and rapid. These circumstances serve to explain the different characters of the illness that attended the suppression of bile in the different cases related. They were many of them cases of essentially different diseases, and having merely this one effect, and the consequences of this effect, in common."

The means of distinguishing jaundice that results from suppressed secretion, from jaundice produced by temporary closure of the ducts, is only easy where the affection follows a powerful emotion, or occurs in the course of purulent phlebitis, or in consequence of some known poisoning, or in several members of a family at the same time, or in quick succession. "In all these instances, knowledge of the cause of the disease, or of some peculiar circumstances under which it may have arisen, gives significance to symptoms that would otherwise be vague and ambiguous." Still, where we have no insight of this kind, and where the cause is unknown to us, we have an important guide in the circumstance that this form of hepatic disease is almost always attended by *diminution in the size of the liver*, while in most other cases of jaundice that organ is enlarged.

Active purging is the treatment which the author considers best adapted to this form of liver complaint; but until more is known respecting its causes, and while the symptoms are so obscure, we are not likely to have satisfactory proofs of the good or ill effects of any particular plans.

In the succeeding section we have described, Fatty degeneration of the liver—Partial deposit of fat in that viscus—Waxy liver—and the appearances caused by deficiency of fat in the liver. Of these, fatty degeneration is the most common. Fat is natural to the liver, but in the healthy state of the organ the quantity is very small. In the fatty liver it is enormously increased. The hepatic cells are gorged with large globules, and the quantity of oil thus accumulated in a liver may equal in weight, and more than equal in bulk all the other elements of the liver put together. The author relates that Vauquelin obtained from a portion of fatty liver, by boiling, as much as 45 parts of oil in 100 of liver. Nearly half the liver, in weight, consisted of uncombined oil. The fatty liver is thus clearly described:

"A liver that has undergone the fatty degeneration, may be little altered in shape, but it is larger, and paler, and softer, and more greasy, than natural. These changes in its sensible qualities depend chiefly, if not solely, on the interstitial deposit of the oil-globules, and their degree may give us some estimate of the quantity of oil the liver contains. When this is very large, the liver is large in proportion, sometimes twice its natural size, and is generally somewhat altered in shape, being thicker than natural, and having its edges blunter or more rounded. The capsule of the liver is stretched and smooth, and when divided its edges recede. The tissue of the liver is pale, and, generally, throughout of a soft buff color, dotted with brown or red. The brown or red dots mark the centres of the lobules, which are usually large and distinct, and are buff-colored near their margins. The liver is very soft, and greases the hands, or the scalpel, like common fat."

The liver undergoes this degeneration in very different states of the body. Thus it occurs in persons who lead indolent lives, and are at the same time gross feeders, and who from these circumstances grow fat. But it is often found in persons dead of phthisis, and of emaciated frames. Its frequency in consumptions was first pointed out by Louis in his celebrated work on phthisis. He found it present in 40 cases out of 120 of that disease. It seems to be four times as frequent in women as in men. Dr. Home found the liver fatty in ten, and *waxy* in five cases, out of sixty-five persons who died of phthisis; and these fifteen instances occurred, with one exception, in females. Fatty degeneration of the liver in such degree as to be at once recognised is almost peculiar to pulmonary consumption, for while it may be detected by the microscope, or by the practised eye in other diseases, it is only in phthisis that it is easily distinguished by sight as a general rule. But this is not invariable; for in some cases of cancerous ulceration the same condition of the liver is presented, and also now and then in ulceration of the large intestine from chronic dysentery. What is the cause of this degeneration of the liver? Baron Larrey advanced the opinion some years ago, that it results from solution of the fat previously laid up in the body, in a manner analogous to what takes place in geese treated so as to develop their livers, according to the following method copied from him:

"To procure the large livers of geese, for the making of patties, fatted birds are confined in close cages, and then exposed to a graduated heat, being kept at the same time entirely without food, even without water. They become feverish, the fat undergoes a kind of fusion, and the liver grows enormously large. The liver is considered to be in the desired state, when the animal is *extremely wasted*, and the fever increases."

The author inclines to this opinion. The liver, upon this supposition, cannot be considered as essentially the seat of the disease, any more than diabetes can be considered a disease of the kidneys. These organs merely eliminate the excess of fat and saccharine matter contained in the blood. The latter substance being soluble passes out of the system, leaving the kidneys unaltered; while the oil-globules are pent up in the liver, adding to its size, and altering its texture.

Another question is presented in view of the details given on this subject. Why does the liver become fatty so much more frequently in women affected with phthisis, than in men? One reason may be, that women, as a general rule, are fatter than men, but it must be confessed that the question cannot be satisfactorily answered.

In some cases the fatty condition of the liver is not general, but confined to a small portion. The author mentions one where a deposit of fat as large as a walnut was found, the rest of the liver being quite sound. These cases depend upon local causes affecting the nutrition of a portion of the liver. and not upon a general cause such as phthisis.

This degeneration of the liver in consumptives may be recognised during the life of the patient; but as it causes but little inconvenience in itself, and leads neither to inflammation nor any other secondary mischief, it is not an object of treatment, especially as the disease with which it is associated is inevitably fatal. When it is the effect of gross feeding, the excess of fat will disappear from the liver, as from other parts, on the individual adopting an abstemious mode of life.

*A scrofulous enlargement of the liver*, to which the next section is devoted, is sometimes met with in persons much wasted by scrofula of the glands or bones, but the matter deposited is different. A similar enlargement is stated by Rokitansky to be produced occasionally by prolonged attacks of ague; but such cases will generally be found complicated with a scrofulous taint. But now and then enlargement takes place without our being able to trace it to scrofula, ague, syphilis, or any of the circumstances to which it is ascribed.

The treatment in this affection of the liver must have reference to the cause. Diet, clothing, sea-air and bathing promise most in cases where it is referrible to scrofula. In such cases the author also thinks well of frictions of iodine ointment long continued. The original malady being faulty assimilation, if we can remedy this, the author thinks we shall probably, in most cases, if not in all, remedy the unnatural condition of the liver, and other secondary affections. At all events, as warned by Dr. Graves, we are not to give up cases of enlargement of the liver too hastily, since sometimes the most unpromising terminate in recovery.

*Excessive and defective secretion of bile*, and *unhealthy states of the bile* are the subjects next discussed.

The quantity of bile secreted varies with the state and circumstances of the individual, and cannot be considered morbid unless secondary disorders are induced by it. In the slighter degrees of this bilious disorder, there is purging of bilious matter, scalding of the rectum, with slight sickness, bitter taste, and a foul tongue, but without much fever. This state of things is readily corrected by an emetic or purgative. In more severe forms of the ailment, fever, pain and tenderness in the region of the liver, and an altered bilous complexion are superadded symptoms. The illness bears a close resemblance to a slight attack of bilious fever. Bleeding may be embraced in the plant of treatment in such cases, and with calomel and saline purgatives will soon correct the disorder. Our author remarks judiciously—

"The readiness with which these attacks are removed, often makes people regard them lightly; but they are important, as evidence of disorders, which, aggravated by time and by continuance in the habits under which they have arisen, may end in some organic disease, or in the total failure of those assimilating processes on which nutrition depends. During the attacks, signal relief is produced by a dose of calomel, or blue pill, followed by saline purgatives. If there should be pain, or tenderness, in the region of the liver, and the patient can well bear it, blood should be taken away by leeches, or by cupping. These measures are generally sufficient for the time, but they do not strike at the root of the evil. Exemption from future attacks, and from the manifold and greater evils to which these disorders may lead as age advances, can only be procured by a change of habits. One of our objects in directing this should be to increase the amount of oxygen inspired, and thus to consume in respiration, or burn off, materials that would otherwise be left for the liver to excrete. The means most efficacious for this purpose, are sea-voyages, riding, or other exercise in the open air, well-ventilated rooms, early rising, the cold or shower-bath, &c. Too much indulgence in sleep, which so much reduces the activity of both respiration and circulation, must be especially injurious, more particularly in rooms that are ill-ventilated, as most bed-rooms are."

It is of equal importance to reduce the diet of the individual in such cases. Butter, fat animal food, and especially spirituous drinks ought to be proscribed.

The other form of this disorder—*diminished secretion of bile*—is marked by indigestion, in its mildest stage, and when more decided is attended by symptoms of jaundice. In both the treatment is the same, and is that which American physicians have long, and, no doubt, too indiscriminately resorted to in all affections of the liver—mercurial cathartics.

The bile may not only be morbid in quantity, but doubtless also altered in quality, although we are not possessed of much knowledge furnished by analyses of human bile. Chemistry has done little more than teach us that the hepatic secretion is sometimes acid; that at others it contains urea; that some medicines pass off along with it; and that some of its constituents are subject to a change in quality. The latter are recognised by the senses, the bile being too light-colored, or too dark-colored, too thin, or too viscid, and, now and then, containing sparkling scales of cholesterine. This ingredient is apt to lead to the formation of *gall-stones*, to which the next section is devoted.

"The shape and size, and appearance of gall-stones varies very much, according to the circumstances under which they are formed.

"When there is only one gall-stone in the bladder, it may grow to the size of a hen's egg, but is seldom found so large. While it remains small, and can move freely in the bladder, it is generally spherical, but when it becomes so large that it is girthed by the bladder, or can no longer roll freely in it, it grows most at the ends which are not subject to pressure, and so becomes somewhat egg-shaped.

"Large solitary gall-stones, with the exception of their nuclei, are composed almost entirely of cholesterine, and are, consequently, white and crystalline. They have a soapy feel, and when placed in the flame of a candle, readily melt, and burn with a bright flame. Sometimes the cholesterine is deposited quite pure, and the gall-stone is then quite white, like a ball of camphor, or of white marble. The surface is generally a little rough and dull, but it readily takes a fine polish. When these round or oval stones are sawn through the centre, they are seen to be crystallized in rays, which converge towards the nucleus.

"A circumstance that seems almost necessary to the formation of gall-stones, is the presence of a small mass of solid biliary matter, or inspissated bile cemented by mucus, or some other substance, about which the cholesterine may collect. Almost all gall-stones have a nucleus, not of cholesterine, which must of course, have existed before them. An excess of cholesterine is not, of itself, sufficient for the formation of gall-stones."

But other elements than biliary occasionally enter into the composition of gall-stones. Our author speaks of *chalky concretions*, composed chiefly of the phosphate or carbonate of lime, as being found in the ducts, gall-bladder, or substance of the liver. He quotes a case from Andral, in which three small calculi of phosphate of lime in the gall-bladder of a man 50 years of age led to his death. Other cases are on record, and Dr. Budd states that in the livers of sheep such chalky concretions are by no means rare.

As in fatty degeneration of the liver, females appear to be the greatest sufferers here. It is the experience of Dr. Prout, whose admirable work on the stomach, we noticed a year ago, that four or five cases of gall-stones occur in women for one in men, and the observation of our author has brought him to the same conclusion. These bodies produce various effects upon the bladder and ducts. One of these is closure of the cystic duct, by which the office of the gall-bladder is destroyed, and indigestion brought about; but as the future passage of gall-stones is thereby prevented, the author thinks the patient may gain by the obstruction. The subject is continued in the following passage which, notwithstanding its length, we are induced, on account of its great interest, to extract:

"If the gall-stone pass through the cystic duct, it generally passes also through the common duct, which is larger and straighter than the cystic duct. If it pass slowly, and be large enough completely to block up the duct and prevent the flow of bile into the intestine, it soon causes jaundice and dilatation of the gall-ducts behind, and of the gall-bladder. The distension of the gall-bladder may be so rapid, and so great, that, on some trifling effort, as that of coughing or of vomiting it may burst, especially if its coats were previously diseased,—and its contents be poured into the cavity of the peritoneum. Several instances of this kind have been recorded. The gall-stone may also become *fastened* in the common duct, and may lead to permanent closure of the duct below it, by adhesion, and consequently, to permanent jaundice and all the other evils which obliteration of the common duct occasions. Sometimes, a large gall-stone gets permanently lodged in the lower end of the common duct, without completely closing it. That part of the duct which embraces the stone, participates in the dilatation of the ducts behind, and bile still passes round the stone into the intestine. This, however, can scarcely happen without much impeding the flow of this fluid, and leading to occasional jaundice, and, in the end, to great dilatation of the hepatic gall-ducts, and greater or less destruction of the secreting element of the liver. But, as before remarked, a gall-stone seldom rests long in the common duct. After a time, which seldom extends beyond a few days, it passes into the intestine. One is occasionally surprised, considering the natural size of the common duct, at the large size of a gall-stone, which has passed through the ducts, without ulceration, into the intestine. A stone, as large as an almond, or larger, may escape in this way. The circumstance shows to what an extent the ducts may be dilated by a constant, and gradual increasing, fluid pressure. When the ducts have been much dilated, they return to their natural size very slowly. The common duct has been found as large as the finger, or even larger, a considerable time after the passage of the stone by which its dilatation was caused."

Inflammation may be excited in the coats of the ducts by the lodgment of gall-stones. At other times, fatty degeneration of the coats seems to be the effect of their mechanical irritation. Again, ulceration of the bladder and ducts results from their presence; and this, in turn, may lead to hepatic abscess, though such a result is probably rare. But long as is this train of evils, flowing from these deposits in the liver, we are not to conclude that their existence is invariably marked by such suffering. On the contrary, according to our author—

"Gall-stones may exist in the *bladder* a long time, without giving rise to any symptoms that are noticed. They are frequently found, and sometimes in great numbers, in persons who during life had no ailment referrible to the liver that could lead one even to suspect them. While stationary in the bladder they give rise to no symptoms, unless they are so large, or so numerous, as to distend it, or unless there be at the same time ulceration or inflammation of its coats. In such cases, they cause a sense of weight or uneasiness in the region of the gall-bladder, or pain in that part, which is felt chiefly after meals, and which sometimes extends through to the right shoulder-blade, or even to the right arm. The pain or uneasiness is increased by a deep breath, or by certain movements of the body.

"The fact that gall-stones often exist without causing pain is explained by the circumstance that the gall-bladder does not contract on the stones, and is perhaps seldom completely emptied, and that gall-stones are so light that they are suspended in bile, and in consequence exert no pressure on the coats of the bladder by reason of their *weight*. It may also be owing in part to the little sensibility to pain which the gall-bladder has when not inflamed."

The next chapter treats of *cancer*, and *hydatids* of the liver, and is full of instruction. The first is necessarily fatal; the treatment can only be palliative. Hydatids are sometimes cured by an effort of the constitution, which is made in one of two ways. The first is by a secretion of a thick matter, like putty, within the sac, by which the tumor is made to grow less, and ceases to disturb the system.

"The second mode of termination—the opening of the tumor and the discharge of its contents through the walls of the belly, or through the intestinal canal, or through the lung—is often followed by obliteration of the sac, disappearance of the tumor, and complete recovery; but it is not unattended with danger. As before remarked, by the admission of air, or otherwise, suppurative inflammation may be set up within the sac, the discharge of the natural contents of the sac may be followed at the end of some days by the discharge of pus, which may continue so as to exhaust the strength of the patient. The probability of a favorable result from such an opening is greater the younger the patient and the more recent the tumor—or rather, the greater the elasticity of the walls of the sac. It is the elasticity of the walls of the sac that closes the cavity as its contents escape and prevents any subsequent mischief."

Common salt, and iodide of potassium are mentioned by our author as articles supposed to have the power of arresting the growth of hydatid tumors.

*Jaundice* is the subject of the concluding chapter in this interesting and instructive volume. This, the author remarks, is a mere symptom which may occur in most diseases of the liver, but then it is a symptom so striking, and an element of so much moment in any case in which it may happen, that a separate consideration of it is required. It may be produced in two ways—by an impediment of the flow of bile into the bowel, and its absorption; or by defective secretion on the part of the liver, the bile not being separated from the blood.

In the greater number of cases the cause is to be found in the latter condition. Often where death has resulted from jaundice no bile has been discovered in the gall-bladder and ducts, showing that the secretion had become null. When this is the case, the bile is eliminated in most of the secretory fluids. It passes off with the urine, with the perspirable matter, the tears, the milk, the mucus and serum of the several passages and cavities. The skin, next to the liver, becomes the most deeply jaundiced, which the author thus accounts for:

"The deep jaundice of the skin doubtless depends on the biliary matter being separated from the blood by the secreting cells of the skin, as it is naturally by those of the liver. The skin and the liver are allied in their office:—both excreting superabundant fatty matter. Many of the constitutional states that favor the secretion of the one, favor that of the other. The skin becomes, thus, in some measure, an index of the manner in which the functions of the liver are performed. Horse-exercise, which clears the skin, clears the liver; mercury, our most effective cholagogue, is excreted in large quantity by the skin."

For the most part, jaundice is attended by bad digestion, impoverished blood, and emaciation; but the author quotes cases from Graves and Stokes in which, after the onset of the disease, "the derangement of the digestive organs subsided, the appetite returned, the bowels became regular, although the stools did not contain a particle of bile, and nutrition continued unimpaired, although the disease had in one case lasted for eight months, and in the other for two years." These facts teach us, that we have attached too much importance to the bile as an agent in digestion; they prove, at least, that a soluble state of the bowels, and adequate nutrition may be maintained when this secretion is deficient, if not entirely shut out from the bowels. The author mentions a man "who was pretty well nourished after four years of jaundice, during which the flow of bile into the intestine seemed to have been completely prevented."

In the worst cases of jaundice, delirium and coma are prominent and fatal symptoms. They depend, says the author, not merely "on the secretion of bile being suspended, but on the poisoned state of the blood, or on the rapid disorganization of the liver, by which the suppression of bile is caused."

The causes of jaundice are very various; obstruction in the gall-ducts, as by a gall-stone passing, cancerous disease of the liver, or inflammation from drink, constipation, pregnancy, spasm of the gall-ducts, are among them. But in the greater number of cases the affection results from the secretion of bile being suppressed or deficient, the causes of which again are various; but we shall not stop to recite them.

"Since, then, jaundice may arise from such various causes, and be a symptom in diseases so different, it is clear that we cannot foretell its issue in any given case, or have well-grounded confidence in our treatment, unless we can pass from the jaundice to the particular disease of the liver on which it depends, or to the particular cause by which it is produced."

In many cases, this is done without difficulty, the symptoms present at the time, or the previous history pointing to the precise condition of the liver of which the jaundice is symptomatic. In respect to the treatment, the author proposes nothing new.

An exceedingly interesting chapter on the *liver-fluke* in sheep is appended to the volume, this parasite being the cause of the *rot* in that animal. Modern researches show that it is also an occasional resident in the human liver, though so rarely that Dr. Budd is not willing to regard it as a cause of disease.

Here we close our extended notice of this work, and in doing so we feel no fear that our readers will charge us with having made it too long. It is far the most valuable contribution made in latter years to our knowledge concerning liver diseases, and will not be esteemed the less so, because it abates somewhat the universality of such disorders. This, undoubtedly, will be its effect. It gives positive information touching the morbid states of the liver, about which there has been so much speculation, and when we have learned what are the reliable signs of disease in it, we shall not be so prone to make it the fountain of every vague disorder.

